# Rapidly progressive antineutrophil cytoplasm antibodies associated with pulmonary-renal syndrome in a 10-year-old girl

**DOI:** 10.1590/S1516-31802001000100008

**Published:** 2001-01-02

**Authors:** Fermin Blanco, Luci Carla Ernesto, Mônica Assis Rosa, Luis Antônio Stuginski, Eliana Regina Zlochevsky, Fernando Blanco

**Keywords:** Pulmonary-renal syndrome, ANCA, Vasculitis, Síndrome pulmonar-renal, ANCA, Vasculite

## Abstract

**CONTEXT:**

The term pulmonary-renal syndrome has been used frequently to describe the clinical manifestations of a great number of diseases in which pulmonary hemorrhage and glomerulonephritis coexist. The classic example of this type of vasculitis is Goodpasture's syndrome, a term used to describe the association of pulmonary hemorrhage, glomerulonephritis and the presence of circulating antiglomerular basement membrane antibodies (anti-GBM). Among the several types of systemic vasculitides that can present clinical manifestations of the pulmonary-renal syndrome, we focus the discussion on two types more frequently associated with antineutrophil cytoplasm antibodies (ANCA), microscopic polyangiitis and Wegener's granulomatosis, concerning a 10 year old girl with clinical signs and symptoms of pulmonary-renal syndrome, with positive ANCA and rapidly progressive evolution.

**CASE REPORT:**

We describe the case of a 10-year-old girl referred to our hospital for evaluation of profound anemia detected in a primary health center. Five days before entry she had experienced malaise, pallor and began to cough up blood-tinged sputum that was at first attributed to dental bleeding. She was admitted to the infirmary with hemoglobin = 4 mg/dL, hematocrit = 14%, platelets = 260,000, white blood cells = 8300, 74% segmented, 4% eosinophils, 19% lymphocytes and 3% monocytes. Radiographs of the chest revealed bilateral diffuse interstitial alveolar infiltrates. There was progressive worsening of cough and respiratory distress during the admission day, when she began to cough up large quantities of blood and hematuria was noted. There was rapid and progressive loss of renal function and massive lung hemorrhage. The antineutrophil cytoplasm antibody (ANCA) test with antigen specificity for myeloperoxidase (anti-MPO) was positive and the circulating anti-GBM showed an indeterminate result.

## RESUMO

**CONTEXTO:**

O termo síndrome pulmonar-renal tem sido usado com freqüência para descrever as manifestações clínicas de um grande número de doenças, nas quais coexistam hemorragia pulmonar e glomerulonefrite. O exemplo clássico desse tipo de vasculite é a síndrome de Goodpasture, um epônimo usado para descrever a associação de hemorragia pulmonar, glomerulonefrite e a presença de anticorpos circulantes anti-membrana basal glomerular (anti-GBM). Dentre os vários tipos de vasculites sistêmicas que podem apresentar as manifestações clínicas da síndrome pulmonar-renal, focalizamos a discussão nos dois tipos mais freqüentemente associados aos anticorpos anti-citoplasma de neutrófilos (ANCA), poliangiite microscópica e granulomatose de Wegener, a propósito de uma menina de 10 anos com sinais e sintomas clínicos da síndrome pulmonar-renal, com ANCA positivo e evolução rapidamente progressiva.

**RELATO DE CASO:**

Nós relatamos o caso de uma menina de 10 anos de idade encaminhada ao nosso hospital para avaliação de anemia profunda detectada em um centro primário de saúde. Cinco dias antes da internação iniciou quadro de tosse, hipoatividade, palidez cutânea e alguns escarros com raias de sangue que foram atribuídos a sangramento dentário. Internada na enfermaria com hemoglobina = 4mg/dl, hematócrito = 14%, plaquetas = 260.000, leucócitos = 8.300, 74% segmentados, 4% eosinófilos, 19% linfócitos e 3% monócitos; Rx tórax com infiltrado interstício alveolar bilateral. Houve piora progressiva da tosse e do desconforto respiratório no mesmo dia da internação, quando apresentou hemoptise maciça e hematúria com evolução rapidamente progressiva para falência renal e hemorragia pulmonar de difícil controle. A sorologia para anticorpos anti-citoplasma de neutrófilos (ANCA), com especificidade anti mieloperoxidase (anti-MPO), foi positiva e o anti-GBM apresentou valor considerado indeterminado.

## INTRODUCTION

The term pulmonary-renal syndrome has frequently been used to describe the clinical manifestations of a great number of diseases in which pulmonary hemorrhage and glomerulonephritis coexist. It consists of a group of complex and often severe disorders that, although rare in incidence, may occasionally be encountered in an Intensive Care Unit.

The classic example of this type of vasculitis is Goodpasture's syndrome, a term used to describe the association of pulmonary hemorrhage, glomerulonephritis and circulating antibodies against glomerular basement membrane (anti-GBM).^1^

In 1958, Stanton and Tange^2^ suggested the term Goodpasture's syndrome to describe a series of young male patients with pulmonary hemorrhage and glomerulonephritis, quite similar to the patient described by Goodpasture in 1919.^3^ It was not until the 1980's, when assays for antineutrophil cytoplasm antibodies (ANCA) were developed, that an association with systemic vasculitides was established.^4,5^ Among the several types of systemic vasculitides that can present clinical manifestations of the pulmonary-renal syndrome, aside from Goodpasture's syndrome, are included: systemic lupus erythematosus, Henoch-Schonlein purpura, mixed cryoglobulinemia and more frequently, Wegener's granulomatosis and microscopic polyangiitis.^6^ We focus the discussion on two types of vasculitides more often associated with antineutrophil cytoplasm antibodies (ANCA), microscopic polyangiitis and Wegener's granulomatosis, concerning a 10 year old girl with signs and symptoms of pulmonary-renal syndrome, with positive ANCA antimyeloperoxidase (anti-MPO) and rapidly progressive evolution.

Even though ANCA and anti-GBM assays are not routinely available in our laboratories, it is crucial to have an early diagnosis of the disorders responsible for pulmonary-renal syndrome because of the danger of rapid, irreversible renal failure or fatality from massive lung hemorrhage.

## CASE REPORT

A 10-year-old white girl believed to have been in good health previously, was admitted to the Pediatric Intensive Care Unit after an episode of massive hemoptysis and respiratory distress. She was referred to our hospital for evaluation of profound anemia detected in a primary health center.

Five days before entry she had experienced malaise, pallor and began to cough up blood-tinged sputum, at first attributed to dental bleeding. She was admitted to the infirmary with hemoglobin = 4 mg/dL, hematocrit = 14%, platelets = 260,000/mm^3^, white blood cells = 8300/mm^3^, 74% segmented, 4% eosinophils, 19% lymphocytes and 3% monocytes. Radiographs of the chest revealed bilateral diffuse interstitial alveolar infiltrates. After laboratory examinations were done, she received packed red blood cells and intravenous cefalotin was started. There was progressive worsening of the cough and respiratory distress during the admission day, when she began to cough up large quantities of blood and was transferred to the Pediatric Intensive Care Unit.

Her past medical history and the family history were noncontributory.

The physical examination upon admission revealed a pale 4+/4+ girl weighing 33 kilogram and 138 cm tall, (NCHS weight by length between 50th and 75^th^ percentiles), Tanner stage 1, tachypneic 2+/4+, dyspneic 2+/4+ with intercostal and subdiaphragmatic retractions, temperature 37 °C, pulse 118/minute, respiratory rate 60/minute, blood pressure 120/60 mmHg, pulse oxymetry 98%, normal heart sounds, no murmurs. Diffuse, coarse rhonchi and crackles were heard in both lung fields. The liver was palpable 2 cm below the right costal margin. There was no edema, cyanosis or signs of deep vein thrombosis, nor cutaneous lesions that could suggest the presence of vasculitis.

Laboratory studies showed a prothrombin time of 14.8 seconds, partial thromboplastin time of 33.6 seconds; hemoglobin 8.0 g/dL; hematocrit 24% after blood transfusion while at the infirmary; serum sodium 136 mEq/L; potassium 5.6 mEq/L; blood urea nitrogen 163.2 mg/dL; serum creatinine 6.13 mg/dL; urinalysis showed specific gravity of 1010, pH 5.0, protein 2+, occult blood 2+, WBC 8500/mL, RBC 240,000/mL, casts were not observed; negative direct and indirect Coombs tests; normal hemoglobin electrophoresis; negative anti-HIV. Laboratory assays for antineutrophil cytoplasm antibodies (ANCA) revealed indirect immunofluorescence for pANCA (perinuclear) with titer 1:80, and cANCA (cytoplasmic) negative. The enzyme linked immunosorbent assay (ELISA) anti-myeloperoxidase (p-ANCA, anti-MPO) was positive and the ELISA anti-GBM result was 6.0 UE/mL.

Serial radiographs of the chest revealed a diffuse increase in bilateral interstitial alveolar infiltrates. There was rapid and progressive worsening of the breathing pattern with massive pulmonary hemorrhage and mechanical ventilation was started. Massive bleeding came up from the tracheal tube during aspiration procedure and she presented a cardiac arrest. Cardiopulmonary resuscitation was successfully achieved after one epinephrine dose. Arterial blood gases drawn fifteen minutes after resuscitation showed pH 6.8, PaCO_2_ 45 mmHg, PaO_2_ 110 mmHg, and bicarbonate 8.6 mEq/L. The patient remained in shock despite the administration of fluid volume, vasoactive drugs and packed red cells. The renal function deteriorated rapidly with oligoanuria and gross hematuria. Peritoneal dialysis was started and the clinical course continued to worsen. Her condition deteriorated fast and she died before the institution of glucocorticosteroids and immunosuppressive therapy.

## DISCUSSION

The systemic vasculitides consist of a broad group of heterogeneous pathologies, whose diagnosis and classification still today remain a great diagnostic challenge.^7,8,9^ Even though there is no universal acceptance, nowadays a tendency exists towards the use of the nomenclature proposed by the International Consensus Conference published in 1994.^10^ According to that classification, the pulmonary-renal syndrome can be divided into three groups:

Little or no immune deposits in the vascular wall (pauci-immune), frequently associated with antineutrophil cytoplasm antibodies (ANCA): Wegener's granulomatosis, microscopic polyangiitis and Churg-Strauss syndrome;

Immune complex deposits: HenochSchonlein purpura, cryoglobulinemia and other types of vasculitides of small vessel such as systemic lupus erythematosus and serum sickness;

Anti-glomerular basement membrane antibody (anti-GBM) deposits: Goodpasture's syndrome.

With the development of assays for antineutrophil cytoplasm antibodies (ANCA), during the 1980's, and the later association with vasculitis (firstly with Wegener's granulomatosis),^11^ this complex group of diseases began to have a laboratory test of great diagnostic value when associated to the clinical signs and symptoms presented by the patient.^4,5,12,13^ These classes of specific autoantibodies against neutrophils and monocytes in cytoplasm present two immunofluorescence staining patterns when the patient's serum is incubated with normal human neutrophils fixed in ethanol. The first pattern produces accentuated immunofluorescence in the central area of neutrophil cytoplasm (cANCA), and is constituted in its great majority by autoantibodies against the enzyme proteinase 3 of the azurophilic granules (anti-PR3); The other pattern, called perinuclear (pANCA), is frequently associated with autoantibodies against myeloperoxidase (anti-MPO), producing perinuclear or nuclear immunofluorescence.^6,14,15^ The positivity of these autoantibodies is fundamental for the differential diagnosis of pulmonary-renal syndrome, firstly because it restricts that group of vasculitides practically to three entities: Wegener's granulomatosis, microscopic polyangiitis and Churg-Strauss syndrome, and also because they are highly sensitive and specific serological markers for those diseases, when serum is tested by indirect immunofluorescence combined with enzyme immunoassays for both PR3 and MPO.^5,12,13,16^ The Churg-Strauss syndrome can be excluded from this discussion because, by definition, it demands the presence of asthma and eosinophilia,^10^ which were not found in the described case.

Although the pathogenic role of antineutrophil cytoplasm antibodies has not yet been proven in these forms of pauci-immune vasculitides, recent evidence suggests that they are directly involved in the mediation of the vascular inflammatory process.^4,17,18^ The finding of antineutrophil cytoplasm antibodies in a patient with manifestations of pulmonary-renal syndrome is highly suggestive of the presence of microscopic polyangiitis or Wegener's granulomatosis.^6^ The ANCA with cytoplasmic pattern (c-ANCA) and antigen specificity for proteinase 3 (anti-PR3) are very sensitive serological markers for Wegener's granulomatosis.

Patients with signs and symptoms of pulmonary-renal syndrome caused by Wegener's granulomatosis present, in their vast majority, positivity for antineutrophil cytoplasm antibodies with c-ANCA pattern anti-PR3.^10^ Patients with microscopic polyangiitis can present positive ANCA with perinuclear pattern (pANCA) anti-MPO or cytoplasmic pattern (cANCA) anti-PR3.

The ANCA-associated pulmonary-renal syndrome, p-ANCA positive with antigen specificity for myeloperoxidase (anti-MPO), is almost always caused by microscopic polyangiitis^10^ and this association can be manifested as severe lung hemorrhage and rapidly progressive renal failure,^15^ as happened with our patient.

It is important to point out that patients with these forms of vasculitides typically present positivity either for c-ANCA anti-PR3 or for p-ANCA anti-MPO, but virtually never for both types.

In addition, the systemic vasculitides that present the association with pulmonary hemorrhage and glomerulonephritis have ANCA circulating more frequently than anti-GBM autoantibodies,^6^ even though, on rare occasions, both can coexist in the same patient.^19,20,21^ Our patient presented an indeterminate result for circulating anti-GBM antibodies (6.0 UE/mL). To be considered positive it should demonstrate a value greater than 20.0 UE/mL.

Bosch et al.^22^ presented a large case series showing that ANCA anti-MPO are highly sensitive and specific markers for glomerular and alveolar necrotizing capillaritis (vasculitis), irrespective of the primary underlying condition. The interpretation of these data allows us to make therapeutic decisions in situations where there is risk to life, in which a histological diagnosis (biopsy) cannot be accomplished. Therefore, the detection of anti-MPO autoantibodies in patients with impaired renal function and lung hemorrhage strongly suggests the presence of glomerular and alveolar capillary vasculitis that, as previously mentioned, can run a rapidly progressive and fatal course.

The clinicopathological diagnosis of the two main types of vasculitis associated with ANCA (Wegener's granulomatosis and microscopic polyangiitis) often overlap, making it difficult to differentiate between them. It is not uncommon to find patients with an initial diagnosis of microscopic polyangiitis (a non-granulomatous inflammation) that later on develop granulomatous inflammation typical of Wegener's granulomatosis.^14,23^

Capillaritis is the most common lung vascular lesion observed in patients with either positive p-ANCA or c-ANCA.^23,24^

Although biopsy is considered the most important technique for the clinicopathological diagnosis of diseases associated with ANCA, it is not uncommon to find specimens without consistent evidence of any specific process. The granulomatous lesions characteristic of Wegener's granulomatosis are frequently only observed after several biopsies.^14^ In rapidly progressive ANCA associated pulmonary-renal syndrome, biopsy is obviously not indicated because of the acute deterioration of the patient's general state.

The clinicopathological differentiation of those patients who show positivity for pANCA anti-MPO is less of a problem, because they only develop granulomatous inflammation on rare occasions.^10^ Therefore it is essential to point out that the important factor lies in the recognition that these autoantibodies are associated with similar pathological processes that can be related to each other and respond basically to the same type of therapy,^18^ namely corticosteroids, immunosuppressors and plasmapheresis.^9^

Another important fact to be borne in mind is that in this particular case, there were signs of poor prognosis from the time of admission to the Intensive Care Unit, such as acute and rapidly progressive onset, massive hemoptysis and serum creatinine greater than 6 mg/dL.^19^

Again we would like to emphasize that the ANCA test results, either positive or negative, should always be analyzed together with clinical manifestations presented by the patient if we want the diagnostic significance of these tests to be optimized.

Jennette, Wilkman and Falk^13^ stated clearly that the patient's signs and symptoms are not just essential when we decide to order the ANCA test but especially when interpreting the significance of the results. We believe that that was an unusual presentation of some type of vasculitis, probably making microscopic polyangiitis the leading possibility clinically.

As stated before, there were many signs of poor prognosis, especially featuring serum creatinine >6 mg/dL at presentation and massive hemoptysis, which substantially increase the mortality rate.^16^

Cases like this, with such an acute presentation, almost invariably run a fatal course even when the immunosuppressive treatment is instituted early.^19^ The clinical course deteriorated so quickly after the admission to the PICU, when the case was presented to us, that we had not enough time to plan and institute the most suitable immunosuppressive treatment. Plasmapheresis was not indicated because she developed a cardiac arrest and remained in shock soon after admission to the PICU.

Our aim in presenting this case is to alert clinicians that, even without the definitive histological diagnosis, it is possible, based on clinical history and physical examination, and whenever possible serological tests (ANCA and anti-GBM), to start immunosuppressive therapy, that can avoid the irreversible loss of renal function and interrupt the fatal course of lung hemorrhage.

We could not finish this discussion without mentioning this providential statement from Jennette et al:^13^ “As with any tool, the usefulness of ANCA testing is dictated by the skill of the individual who uses it.”
